# LncRNA DANCR involved osteolysis after total hip arthroplasty by regulating FOXO1 expression to inhibit osteoblast differentiation

**DOI:** 10.1186/s12929-018-0406-8

**Published:** 2018-01-16

**Authors:** Zhenyu Tang, Zongming Gong, Xiaoliang Sun

**Affiliations:** Department of Articular Orthopaedics, Changzhou First People’s Hospital, The Third Affiliated Hospital of Soochow University, No.185 Juqian Rd, Changzhou, Jiangsu 213003 China

**Keywords:** Total hip arthroplasty, DANCR, FOXO1, Osteoblast differentiation

## Abstract

**Background:**

Aseptic loosening of artificial hip joint is a major complication affecting the long-term use of the artificial hip joint, and is the main cause of joint replacement failure. However, the mechanism of aseptic loosening of THR has not yet cleared. The aim of this study was to investigate the underlying mechanism of DANCR in osteoblast differentiation (OD).

**Methods:**

We detected the expressions of DANCR and FOXO1 in clinical samples and mesenchymal stem cells (MSCs) by qRT-PCR and western blotting. The effects of polymethylmethacrylate (PMMA) on OD of MSCs were examined by alkaline phosphatase (ALP) activity and Alizarin Red S (ARS) staining. The expressions of OD markers were measured by qRT-PCR and western blotting. The mechanism of DANCR in OD was detected by RNA pull-down, RNA immunoprecipitation (RIP) assay and ubiquitination assays.

**Results:**

Compared with the surrounding normal tissues, DANCR expression was up-regulated and FOXO1 expression was down-regulated in periprosthetic tissues. PMMA suppressed ALP activity, increased DANCR expression, and decreased the expressions of FOXO1, Runx2, Osterix (Ostx) and osteocalcin (OCN). ARS staining showed that PMMA inhibited the OD of MSCs. Knockdown of DANCR attenuated the inhibitory effect of PMMA on OD. Knockdown of FOXO1 could reverse the effect of si-DANC. RNA pull-down and RIP assay implicated that DANCR bound to FOXO1. Ubiquitination assay indicated that si-DANCR could repress Skp2-mediated ubiquitination of FOXO1.

**Conclusion:**

LncRNA DANCR could inhibit OD by regulating FOXO1 expression.

## Background

Total hip replacement (THR) is one of the most common methods for the treatment of osteoarthritis, rheumatoid arthritis and femoral neck fracture in the elderly. It can effectively relieve joint pain, improve joint function and elevate the quality of life of patients [1]. Aseptic loosening of artificial hip joint is a major complication affecting the long-term use of the artificial hip joint, and is the main cause of joint replacement failure. Prosthesis wear particles stimulate the macrophages around the prosthesis to produce a variety of cytokines, which can activate osteoclasts to enhance bone absorption, affect osteoblast differentiation (OD), and inhibit bone formation [2]. However, the mechanism of aseptic loosening of THR is complicated, and it has not yet cleared.

Long non-coding RNA (lncRNA) is a class of RNA greater than 200 nt in length that does not have the ability to translate into proteins [3]. Studies have proved that some lncRNAs can play an important role in regulation of epigenetic level, transcriptional level, translational level and protein modification [4, 5]. Recently, lncRNA has been found to be involved in OD. Zhu et al. [5] reported that lncRNA ANCR could inhibit OD via regulating Runx2. Huang et al. [6] revealed that lncRNA H19 promoted OD by miR-675/TGF-β1/Smad3/HDAC pathway. LncRNA DANCR, a highly expressed lncRNA in tumor, was first discovered by Kretz et al. [7] in 2012. It has been reported that DANCR is involved in the differentiation of many cells, such as hepatoma cells, osteoblasts and cartilage cells [8, 9]. Zhang et al. [10] found that DANCR promoted chondrogenic differentiation through up-regulating the expression of Smad3 and STAT3. However, the relationship between DANCR and OD has not been reported.

Fork box transcription factor (FOXO) is a subclass of the fork box protein family, which plays a major regulatory role in many biological processes [11]. Studies have found that FOXO family is related to OD and bone formation. Moriishi et al. [12] found that Bcl2 could promote OD via activate FOXO. Iyer et al. [13] reported that FOXO inhibited bone formation by repressing Wnt signaling. FOXO1 is one of the important members in FOXO family, which mainly involves in metabolism, cell proliferation, oxidative stress (OS) and cell death [14]. It has been found that FOXO1 plays an important role in OD, bone formation and bone remodeling [15, 16].

In this study, we investigated the underlying mechanism of DANCR in OD. We found that DANCR could inhibit OD by regulating FOXO1 expression, then participate in osteolysis after THR.

## Methods

### Clinical samples

From October 2013 to March 2017, we screened 20 patients (12 males and 8 females, aged from 54 to 80 years old) who were admitted in our hospital and underwent revision of THR because of prosthetic aseptic loosening. Their median age was 66 years. Inclusion criteria: patients have clinical manifestations such as pain and movement disorder, the imaging findings include clear line around the prosthesis and obvious shift of prosthesis. Periprosthetic wear particles and surrounding tissues were collected during revision surgery. The expressions of DANCR and FOXO1 in the clinical samples were measured by qRT-PCR and western blotting. All experimental protocols were approved by the Ethical Committee of Changzhou first people’s hospital.

### Quantitative real-time PCR (qRT-PCR)

According to the manufacturer’s instructions, total RNA was extracted from tissues or cells using Trizol reagent (Invitrogen, CA, USA). Then, cDNAs were synthesized and qRT-PCR for the mRNA levels of DANCR, FOXO1, Runx2, Osterix (Ostx) and osteocalcin (OCN) were conducted using SYBR Green Master Mix (Takara, Japan). GAPDH was used as the internal reference. The primers sequence was as follows:

DANCR--F: 5’-GCCACTATGTAGAG GGTTTC-3’.

DANCR--R: 5’-ACCTGCGCTAAGAA-CTGAGG-3’.

FOXO1--F: 5’-GGCTGAGGGTTAGTGAGCAG-3’.

FOXO1--R: 5’-AAAGGGAGTTGGTGAAAGACA-3’.

Runx2--F: 5’-ACTTCCTGTGCTCGGTGCT-3’.

Runx2--R: 5’-GACGGTTATGGTCAAGGTGAA-3’.

Ostx--F: 5’-GGCACAAAGAAGCCGTACTC-3’.

Ostx--R: 5’-GCCTTGTACCAGGAGCCATA-3’.

OCN--F: 5’-GGCAGCGAGGTAGTGAAGAG-3’.

OCN--R: 5’-CTGGAGAGGAGCAGAACTGG-3’.

GAPDH--F: 5’-TCGACAGTCAGCCGCATCTTCTTT-3’.

GAPDH--R: 5’-GCCCAATACGACCAAATCCGTTGA-3’.

### Western blotting

Total protein was extracted from tissues or cells using RIPA Lysis Buffer (Beyotime, Beijing, China). The protein was then separated by 12% SDS-PAGE and transferred onto PVDF membranes (Millipore, USA). The membranes were blocked with 5% skim milk and incubated with primary antibodies, including anti-FOXO1 antibody (1:400, Cell Signaling Technology), anti-ALP antibody, anti-Runx2 antibody (1:300, Cell Signaling Technology), anti-Ostx antibody (1:300, Cell Signaling Technology), anti-OCN antibody (1:300, Cell Signaling Technology), anti-Skp2 antibody (1:500, Santa Cruz, USA) and anti-GAPDH antibody (1:800, Santa Cruz). Then, the membranes were incubated with anti-rabbit horseradish peroxidase-conjugate secondary antibody (1:2000, Santa Cruz) at room temperature for 1 h. ECL development solution (Thermo, USA) was used to visualize the proteins.

### Mesenchymal stem cells (MSCs)

MSCs were purchased from Shanghai Jining Biological Technology Co. Ltd. MSCs were incubated in complete medium supplemented with 10% fetal bovine serum (FBS, Gibco) and 100 U/mL penicillin-streptomycin (Invitrogen) at 37 °C in 5% CO_2_. Polymethylmethacrylate (PMMA) was purchased from MITSUBISHI CHEMICAL POLYMER NANTONG CO. Ltd. (Nantong, China). Grouping methods of MSCs: (1) Control group and PMMA group. MSCs in PMMA group were treated with 0.3% *v*/v PMMA. (2) Control group, PMMA group, PMMA+siRNA group and PMMA+si-DANCR group. (3) pcDNA group and pcDNA-DANCR group. pcDNA-DANCR (Invitrogen) was used to over-express DANCR. (4) Control group, PMMA group, PMMA+siRNA group, PMMA+si-DANCR group and PMMA+si-DANCR+si-FOXO1 group. MSCs in each group were incubated in complete medium containing 200 ng/mL bone morphogenetic protein 2 (BMP2; Peprotech, USA) for 14 d. PMMA stimulation was performed at 48 h after transfection with si-DANCR (5’-GGAGCTAGAGCAGTGACAATG-3′) or si-FOXO1 (5’-GAGGAUUGAACCAGUAUAATTUUAUACUGGUUCAAUCCUCTT-3′). The cell transfection was performed using Lipofectamine 2000 (Invitrogen) according to the manufacturer’s instruction. After these treatments, cells were harvested for qRT-PCR or western blot analysis.

### The human embryonic kidney 293 cells (HEK-293 cells)

HEK-293 cells were purchased from Shanghai Jining Biological Technology Co. Ltd. HEK-293 cells were incubated in DMEM medium (Gibico, USA) containing 10% FBS and 100 U/mL penicillin-streptomycin (Invitrogen) at 37 °C in 5% CO_2_. Grouping methods of HEK-293 cells: (1) S-phase kinase-associated protein 2 (Skp2) group and Skp2 + si-DANCR group. Skp2 and si-DANCR plasmids were transiently transfected into cells using jetPRIME (Polyplus Transfection, France) according to the directions of reagent. Twenty-four hours after transfection, 10 μg/mL cycloheximide (CHX) was added into DMEM medium. Cells were incubated in this medium for 0, 3, 6 and 9 h. (2) Ubiquitin (UB) + Skp2 + pcDNA-FOXO1 + MG13 group and UB + Skp2 + pcDNA-FOXO1 + MG132 + DANCR group. A ubiquitination assay was performed in the 2 groups.

### Ubiquitination assay

Ub (Ubbiotech, Changchun, China), Skp2 and DANCR plasmids, and pcDNA-FoxO1 (Addgene, USA) were transiently transfected into cells using jetPRIME (Polyplus Transfection). After 36 h, 10 nm MG132 (Yeasen, Shanghai) was added into DMEM medium. Cells were incubated in this medium for 8 h. After these treatments, cells were harvested for western blot analysis. The cell lysates were immunoprecipitated (IP) with the labeled antibodies at 4 °C overnight. Western blotting was used to detect the eluted proteins.

### Alkaline phosphatase (ALP) activity

According to manufacturer’s instructions, ALP activity detection kit (Yeasen) was used to measure the ALP activity. Briefly, MSCs were lysed with Triton X-100 lysis buffer (Yeasen), and centrifuged at 1000×g for 20 min. ALP activity in the cell supernatant was then measured according to manufacturer’s instructions.

### Alizarin red S (ARS) staining

ARS staining was used to detect mineralization. MSCs were fixed with 4% paraformaldehyde for 10 min, and stained with 1% ARS solution (yuanyebio, Shanghai) at room temperature for 30 min. MSCs were then washed with PBS. The stained cells and mineral nodules were observed at 450 nm in a microplate reader (Thermo).

### RNA pull-down

RNA pull-down was performed using RNA-Protein Pull-Down Kit (Thermo) according to the manufacturer’s instructions. Briefly, T4 RNA polymerase (Roche) was used to label DANCR. Biotinylated RNAs were then mixed with streptavidin magnetic beads (Invitrogen) at 4 °C overnight. The cell lysates were interacted with biotinylated RNA on ice for 1 h. Finally, The RNA-protein binding mixtures were identified by western blot assay.

### RNA immunoprecipitation (RIP) assay

According to the manufacturer’s instructions, RIP assay kit (Millipore, USA) was used for RIP assay. Briefly, HEK-293 cell suspension was prepared in RIP buffer. Anti-FOXO1 antibody (Cell Signaling Technology, 5 μg) was incubated with the cell suspension at 4 °C overnight. Then, the precipitated RNA was purified and analyzed by qRT-PCR. Isotype-matched IgG (5 μg) was used as a negative control.

### Statistical analysis

SPSS 20.0 software was used to calculate the statistical analysis. All data were expressed as means ± standard deviation (SD). Student *t* test or one-way analysis of variance (ANOVA) was used to compare quantitative variables. Differences between groups were considered statistically significant if *P* < 0.05.

## Results

### The expression of DANCR and FOXO1 in periprosthetic wear particles and surrounding tissues

**Fig. 1:**
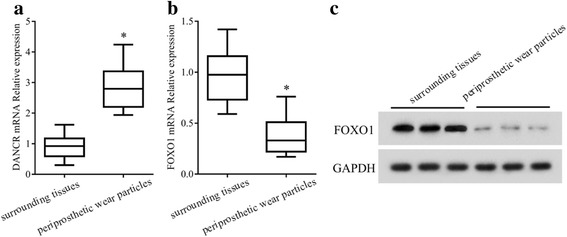
The expression of DANCR and FOXO1 in periprosthetic wear particles and surrounding tissues. Periprosthetic wear particles and surrounding tissues were collected during revision surgery. The expressions of DANCR (**a**) and FOXO1 (**b**&**c**) in the clinical samples were measured by qRT-PCR and western blotting. *P < 0.05, vs normal

To explore the role of DANCR and FOXO1 in osteolysis, we first evaluated their expression levels in periprosthetic wear particles and surrounding tissues. Compared with the surrounding normal tissues, the expression of DANCR (Fig. 1a) was increased and the mRNA (Fig. 1b) and protein (Fig. 1c) expression of FOXO1 was decreased in periprosthetic tissues.

### The effect of PMMA on OD of MSCs

**Fig. 2:**
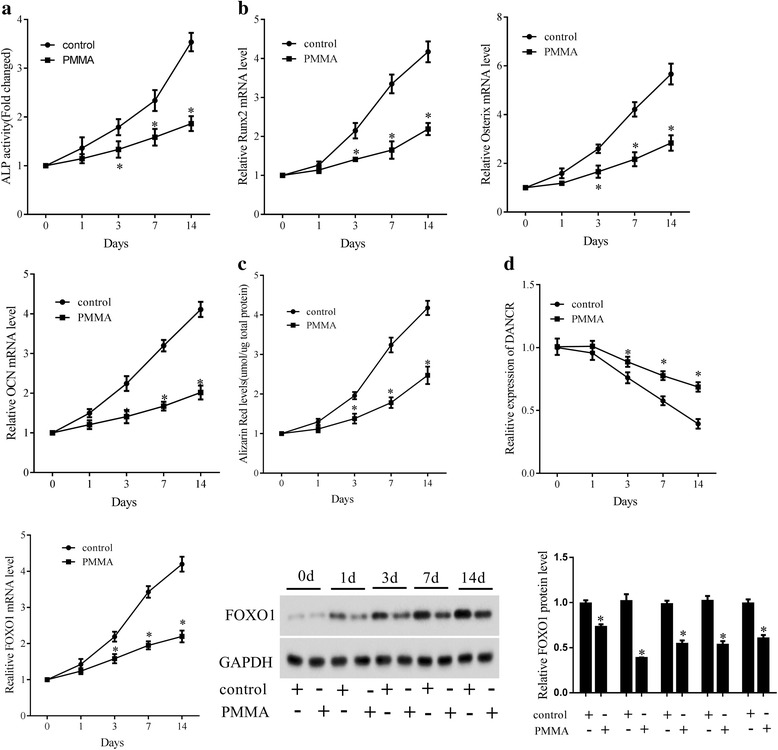
The effect of PMMA on osteoblast differentiation of MSCs. MSCs were divided into control group and PMMA group. The degree of osteoblast differentiation and the levels of OD markers were measured at day 0, 1, 3, 7 and 14. **a** ALP activity. **b** The expression of Runx2, Osterix and OCN. **c** ARS staining. **d** The expression of DANCR and FOXO1. *P < 0.05, vs control

To investigate the effect of PMMA on the OD of MSCs, MSCs were divided into 2 groups (control and PMMA group). The degree of OD and the levels of OD markers were measured at day 0, 1, 3, 7 and 14. Results showed that ALP activity was lower in PMMA group than in control group at each time point (Fig. 2a). qRT-PCR assay revealed that the mRNA expressions of Runx2, Ostx and OCN were down-regulated by PMMA (Fig. 2b). ARS staining showed that PMMA inhibited the OD of MSCs (Fig. 2c). Additionally, PMMA could elevate DANCR expression and reduce FOXO1 expression (Fig. 2d).

### Knockdown of DANCR attenuated the inhibitory effect of PMMA on OD of MSCs

**Fig. 3:**
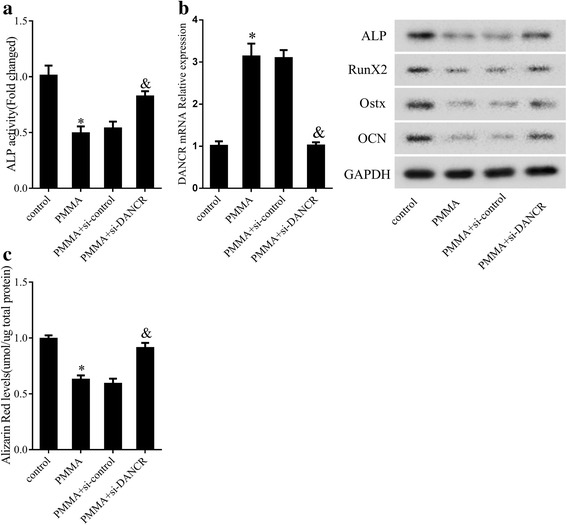
Knockdown of DANCR attenuated the inhibitory effect of PMMA on osteoblast differentiation of MSCs. MSCs were divided into control group, PMMA group, PMMA+siRNA group and PMMA+siRNA DANCR group. **a** ALP activity. **b** The expression of DANCR, ALP, Runx2, Osterix and OCN. **c** ARS staining. *P < 0.05, vs control. ^&^*P* < 0.05, vs PMMA+siRNA

To detect the effect of DANCR on OD, MSCs were divided into 4 groups (control group, PMMA group, PMMA+siRNA group, PMMA+siRNA DANCR group). Results showed that PMMA could inhibit ALP activity, and si-DANCR reversed the inhibitory effect (Fig. 3a). Moreover, PMMA could up-regulate the expression of DANCR, and si-DANCR down-regulated its expression (Fig. 3b). Western blotting results showed that PMMA repressed the expressions of ALP, Runx2, Ostx and OCN, while si-DANCR could reverse the effect of PMMA (Fig. 3b). ARS staining revealed that si-DANCR reversed the inhibitory effect of PMMA on the OD of MSCs (Fig. 3c).

### DANCR directly interacted with FOXO1

**Fig. 4:**
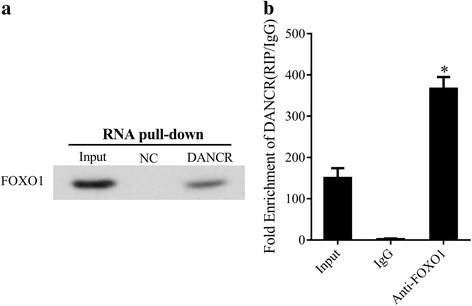
DANCR directly interacted with FOXO1. To investigate the relationship between DANCR and FOXO1, RNA pull-down assay (**a**) and RIP assay (**b**) were used to verify the association of DANCR with FOXO1 in HEK-293 cells. Results showed that DANCR bound to FOXO1. *P < 0.05, vs input

To investigate the relationship between DANCR and FOXO1, we performed RNA pull-down assay (Fig. 4a). Moreover, RIP assay was used to verify the association of DANCR with FOXO1 in HEK-293 cells (Fig. 4b). Results implicated that DANCR bound to FOXO1.

### Knockdown of DANCR inhibited the Skp2-mediated ubiquitination of FOXO1

**Fig. 5:**
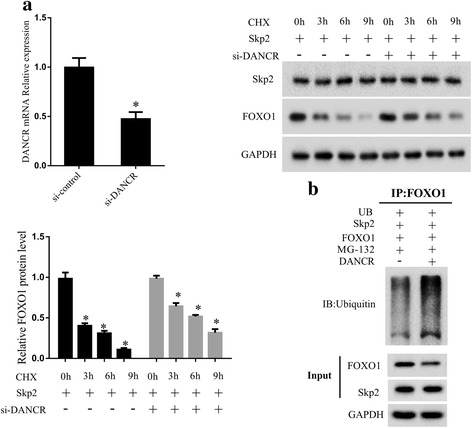
Knockout of DANCR inhibited the Skp2-mediated ubiquitination of FOXO1. **a** CHX chase assay. Compared with cells transfected with Skp2 alone, FOXO1 expression was more stable in cells transfected with Skp2 and si-DANCR. **b** Ubiquitination assay. Results showed a markedly decrease in FOXO1 expression in DANCR transfected cells

Firstly, we carried out a CHX chase assay to detect the effect of DANCR on the stability of FOXO1 protein. Compared with cells transfected with Skp2 alone, FOXO1 expression was more stable in cells transfected with both Skp2 and si-DANCR (Fig. 5a). Then, we performed a ubiquitination assay, which showed a significant decrease in polyubiquitinated FOXO1 protein in Skp2-transfected cells, whereas DANCR overexpression increased FOXO1 ubiquitination (Fig. 5b). The results indicated that knockdown of DANCR could repress Skp2-mediated ubiquitination of FOXO1.

### Effect of DANCR on FOXO1 in MSCs

**Fig. 6:**
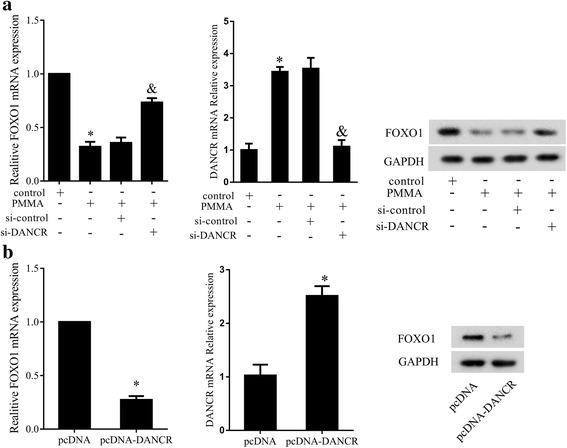
Effect of DANCR on FOXO1 in MSCs. FOXO1 expression was measured by qRT-PCR and western blotting. **a** The effect of si-DANCR on FOXO1 expression. **b** The effect of pcDNA-DANCR on FOXO1 expression. *P < 0.05, vs control or pcDNA. ^&^*P* < 0.05, vs PMMA+siRNA

To detect the effect of DANCR silencing on FOXO1 expression, MSCs were divided into control group, PMMA group, PMMA+siRNA group, and PMMA+si-DANCR group. FOXO1 expression was measured by qRT-PCR and western blotting. Results showed that PMMA repressed the expression of FOXO1 and increased DANCR expression, while si-DANCR could enhance the mRAN and protein expression of FOXO1, and reduce DANCR expression (Fig. 6a). Then, MSCs were divided into pcDNA group and pcDNA-DANCR group. pcDNA-DANCR was used to over-express DANCR (Fig. 6b). Results showed that over-expression of DANCR could reduce FOXO1 expression (Fig. 6b).

### PMMA influenced the OD of MSCs by regulating DANCR/FOXO1

**Fig. 7:**
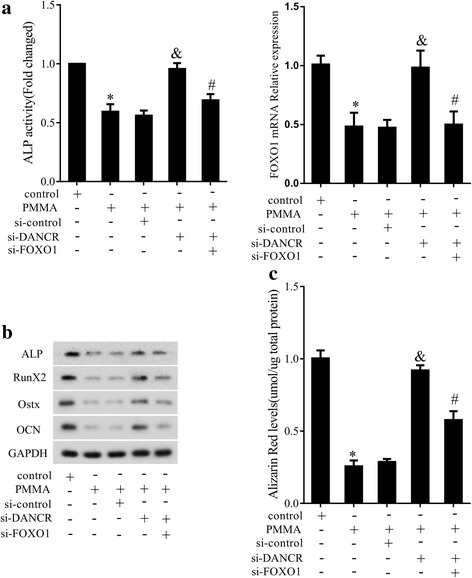
PMMA influenced the osteoblast differentiation of MSCs by regulating DANCR/FOXO1. MSCs were divided into control group, PMMA group, PMMA+siRNA group, PMMA+si-DANCR group and PMMA+si-DANCR+si-FOXO1 group. **a** ALP activity and FOXO1 expression. **b** The expression of ALP, Runx2, Osterix and OCN. **c** ARS staining. *P < 0.05, vs control. ^&^*P* < 0.05, vs PMMA+siRNA. ^#^*P* < 0.05, vs PMMA+si-DANCR

To investigate the mechanism of PMMA on the OD of MSCs, MSCs were divided into control group, PMMA group, PMMA+siRNA group, PMMA+si-DANCR group and PMMA+si-DANCR+si-FOXO1 group. Results revealed that PMMA repressed ALP activity, and knockdown of DANCR could enhance ALP activity. However, knockdown of FOXO1 could reverse the effect of DANCR silencing (Fig. 7a). Moreover, PMMA could down-regulate FOXO1 expression, and si-DANCR up-regulated its expression, and si-FOXO1 reversed the effect (Fig. 7a). Western blotting results showed that the expressions of ALP, Runx2, Ostx and OCN were reduced by PMMA, and were increased by si-DANCR. Knockdown of FOXO1 could decrease the expressions (Fig. 7b). ARS staining showed that PMMA inhibited the OD of MSCs, but knockdown of DANC could reverse the inhibitory effect. Meanwhile, si-FOXO1 could reverse the effect of si-DANC (Fig. 7c).

## Discussion

Nowadays, THR is the major method for the treatment of end-stage hip joint diseases [17], which can increase joint function and improve the quality of life of patients [18]. However, postoperative complications are the greatest threat for patients. Aseptic loosening is the leading cause of postoperative complications, and periprosthetic osteolysis is the major cause of it [19]. At present, no matter what prosthesis used in THR, osteolysis always be probably occurred [20]. Therefore, how to solve the problem of prosthesis loosening after THR has been a research hotspot in recent years.

LncRNAs have been reported to be related with OD and bone formation [21]. Xu et al. [22] found that lncRNA HIF1α-AS1 promote OD through regulating HOXD10. Cui et al. [23] revealed that silencing of lncRNA NONHSAT009968 could attenuate the inhibitory effect of Staphylococcal protein A on OD. Tong et al. [4] pointed out that DANCR might a biomarker for osteoporosis. Moreover, DANCR has been reported to regulate chondrogenic differentiation [8, 10, 24]. The present study found an increased expression of lncRNA DANCR in periprosthetic wear particles and surrounding tissues, which indicated that DANCR might be related to OD. Subsequently, we investigated the effect of DANCR in OD of MSCs. We found that knockdown DANCR could reverse the inhibitory effect of wear particles on OD. Next, we further examined the underlying mechanism of DANCR in OD.

The further research demonstrated that DANCR could bind to FOXO1, and knockout of DANCR could inhibit Skp2-mediated ubiquitination of FOXO1. Skp2 can specifically recognize phosphorylated substrates and mediate their ubiquitination degradation [25]. Skp2 has been reported to target FOXO1 and promote the its degradation [26]. Our research had the similar results. FOXO1, a transcription factor, can regulate cellular proliferation, gluconeogenesis, energy metabolism and OS through transcription and conduction of growth factors and cytokine signaling [14]. In recent years, studies have shown that FOXO1 influences the formation of new bone via regulating the proliferation, differentiation and apoptosis of osteoblasts [15]. Rached et al. [27] found a marked reduction in osteoblast number and bone formation rate, and an increase in bone resorption when knockout of FOXO1 gene in osteoblasts of mice. In addition, MSCs, a source of osteoblasts, are also involved in the process of bone formation. Studies have shown that FOXO1 plays an important role in OD of stem cells and osteoblast precursor cells [28]. The expression of FOXO1 increases when MSCs is differentiated into osteoblasts. Similar findings were also found in our study. Moreover, we found that DANCR could negatively regulate the mRNA and protein expression of FOXO1. We also manifested that DANCR regulated the role of wear particle in OD, and knockdown of FOXO1 had a reversal effect on the regulation.

## Conclusion

In summary, the present study demonstrates that DANCR could inhibit OD by regulating FOXO1 expression. The findings suggested that DANCR might be a potential therapeutic target in osteolysis after THR.

## References

[1] Klugarova J, Klugar M, Gallo J (2016). The effectiveness of inpatient physical therapy compared to outpatient physical therapy for older adults after total hip replacement in the post-discharge period: a systematic review protocol. JBI Database System Rev Implement Rep.

[2] Gallo J, Vaculova J, Goodman SB (2014). Contributions of human tissue analysis to understanding the mechanisms of loosening and osteolysis in total hip replacement. Acta Biomater.

[3] Batista PJ, Chang HY (2013). Long noncoding RNAs: cellular address codes in development and disease. Cell.

[4] Tong X, Gu P, Xu S (2015). Long non-coding RNA-DANCR in human circulating monocytes: a potential biomarker associated with postmenopausal osteoporosis. Biosci Biotechnol Biochem.

[5] Zhu L, Xu PC (2013). Downregulated LncRNA-ANCR promotes osteoblast differentiation by targeting EZH2 and regulating Runx2 expression. Biochem Biophys Res Commun.

[6] Huang Y, Zheng Y, Jia L (2016). Long noncoding RNA H19 promotes osteoblast differentiation via TGF-β1/Smad3/HDAC signaling pathway by deriving miR-675. Stem Cells.

[7] Kretz M, Webster DE, Flockhart RJ (2012). Suppression of progenitor differentiation requires the long noncoding RNA ANCR. Genes Dev.

[8] Zhang L, Chen S, Bao N (2015). Sox4 enhances chondrogenic differentiation and proliferation of human synovium-derived stem cell via activation of long noncoding RNA DANCR. J Mol Histol.

[9] Yuan SX, Wang J, Yang F (2015). Long noncoding RNA DANCR increases stemness features of hepatocellular carcinoma via de-repression of CTNNB1. Hepatology.

[10] Zhang L, Yang C, Chen S (2017). Long noncoding RNA DANCR is a positive regulator of proliferation and Chondrogenic differentiation in human synovium-derived stem cells. DNA Cell Biol.

[11] Ponugoti B, Dong G, Graves DT (2012). Role of Forkhead transcription factors in diabetes-induced oxidative stress. Exp Diabetes Res.

[12] Moriishi T, Kawai Y, Komori H (2014). Bcl2 deficiency activates FoxO through Akt inactivation and accelerates osteoblast differentiation. PLoS One.

[13] Iyer S, Ambrogini E, Bartell SM (2013). FOXOs attenuate bone formation by suppressing Wnt signaling. J Clin Invest.

[14] Puthanveetil P, Wan A, Rodrigues B (2013). FoxO1 is crucial for sustaining cardiomyocyte metabolism and cell survival. Cardiovasc Res.

[15] Siqueira MF, Flowers S, Bhattacharya R (2011). FOXO1 modulates osteoblast differentiation. Bone.

[16] Tan P, Guan H, Xie L (2015). FOXO1 inhibits osteoclastogenesis partially by antagnozing MYC. Sci Rep.

[17] Dargel J, Oppermann J, Brüggemann GP (2014). Dislocation following total hip replacement. Dtsch Arztebl Int.

[18] Dowsey MM, Nikpour M, Dieppe P (2016). Associations between pre-operative radiographic osteoarthritis severity and pain and function after total hip replacement : radiographic OA severity predicts function after THR. Clin Rheumatol.

[19] Mulcahy H, Chew FS (2012). Current concepts of hip arthroplasty for radiologists: part 2, revisions and complications. AJR Am J Roentgenol.

[20] Banaszkiewicz PA (2014). “Modes of failure” of cemented stem-type femoral components: a radiographic analysis of loosening. Classic Papers in Orthopaedics.

[21] Huang G, Kang Y, Huang Z (2017). Identification and characterization of long non-coding RNAs in osteogenic differentiation of human adipose-derived stem cells. Cell Physiol Biochem.

[22] Xu Y, Wang S, Tang C (2015). Upregulation of long non-coding RNA HIF 1α-anti-sense 1 induced by transforming growth factor-β-mediated targeting of sirtuin 1 promotes osteoblastic differentiation of human bone marrow stromal cells. Mol Med Rep.

[23] Cui Y, Lu S, Tan H (2016). Silencing of long non-coding RNA NONHSAT009968 ameliorates the staphylococcal protein A-inhibited osteogenic differentiation in human bone mesenchymal stem cells. Cell Physiol Biochem.

[24] Zhang L, Sun X, Chen S (2017). Long noncoding RNA DANCR regulates miR-1305-Smad 4 axis to promote chondrogenic differentiation of human synovium-derived stem cells. Biosci Rep.

[25] Gstaiger M, Jordan R, Lim M (2001). Skp2 is oncogenic and overexpressed in human cancers. Proc Natl Acad Sci U S A.

[26] Huang H, Regan KM, Wang F (2005). Skp2 inhibits FOXO1 in tumor suppression through ubiquitin-mediated degradation. Proc Natl Acad Sci U S A.

[27] Rached MT, Kode A, Xu L (2010). FoxO1 is a positive regulator of bone formation by favoring protein synthesis and resistance to oxidative stress in osteoblasts. Cell Metab.

[28] Teixeira CC, Liu Y, Thant LM (2010). Foxo1, a novel regulator of osteoblast differentiation and Skeletogenesis. J Biol Chem.

